# Losses in the Care Cascade for Latent Tuberculosis Infection in the Tracing Contact Studies

**DOI:** 10.3390/pathogens12121403

**Published:** 2023-11-29

**Authors:** Sofia Godoy, Ignasi Parrón, Joan-Pau Millet, Joan A. Caylà, Núria Follia, Mònica Carol, Àngels Orcau, Miquel Alsedà, Diana Toledo, Glòria Ferrús, Pere Plans, Irene Barrabeig, Laura Clotet, Àngela Domínguez, Pere Godoy, the Transmission of Tuberculosis in Catalonia (Spain) Working Group

**Affiliations:** 1 Institut de Recerca Biomédica de Lleida (IRBLleida), Universitat de Lleida, 25198 Lleida, Spain; sofiagodoygarcia@gmail.com (S.G.); miquel.alseda@gencat.cat (M.A.); 2 Institut Català de la Salut, 25198 Lleida, Spain; 3 Agència de Salut Pública Catalunya, 08005 Barcelona, Spain; iparron@gencat.cat (I.P.); nfollia@gencat.cat (N.F.); monica.carol@gencat.cat (M.C.); gloria.ferrus@gencat.cat (G.F.); pedro.plans@gencat.cat (P.P.); ibarrabeig@gencat.cat (I.B.); lclotet.mn.ics@gencat.cat (L.C.); 4 Consorcio de Investigación Biomédica en Red de Epidemiología y Salud Pública (CIBERESP), 28029 Madrid, Spain; jmillet@aspb.cat (J.-P.M.); dtoledo@ub.edu (D.T.); angela.dominguez@ub.edu (À.D.); 5 Tuberculosis Research Unit Foundation, 08008 Barcelona, Spain; joan.cayla@uitb.cat; 6 Agència de Salut Pública de Barcelona, 08023 Barcelona, Spain; aorcau@aspb.cat; 7 Departament de Medicina, Universitat de Barcelona, 08036 Barcelona, Spain; 8 Hospital Universitari de Santa Maria, Faculty of Medicine, 25198 Lleida, Spain

**Keywords:** tuberculosis, latent tuberculosis infection, contact tracing, epidemiology

## Abstract

**Background**: The control of latent tuberculosis infection (LTBI) encompasses multiple stages. The objective was to calculate the losses in the LTBI care cascade for pulmonary TB contacts in Catalonia (Spain). **Methods**: The LTBI care cascade was studied for pulmonary TB contacts reported from 1 January 2019 to 30 June 2021, considering three dependent variables: non-performance of testing; non-receipt of a treatment prescription; and non-adherence to treatment. Variables associated with the cascade were analysed using adjusted OR (aOR) and 95% confidence intervals (CI). **Results**: Identified from 847 cases of pulmonary TB were 7087 contacts, of whom 6537 (92.2%) could be screened for LTBI. LTBI prevalence was 25.5% (1670/6537); 69.4% of persons with LTBI (1159/1670) received a treatment prescription and 71.3% (827/1159) completed it. Treatment prescription was associated with age ≥65 years (aOR = 0.3; 95%CI: 0.2–0.6) and a daily exposure of ≥6 h to the TB index case (aOR = 3.6; 95%CI: 2.6–5.0). Treatment adherence was lower in men (aOR = 0.7; 95%CI: 0.5–1.0) and immigrants (aOR = 0.7; 95%CI: 0.5–0.9). **Conclusions**: Under 50% of contacts make it to the end of the LTBI cascade. Losses need to be reduced through education of both healthcare providers and patients and through treatment monitoring. The greater involvement of primary care physicians could help in monitoring and controling LTBI.

## 1. Introduction

The United Nations (UN) and the World Health Organization (WHO), having set ambitious targets to reduce the global burden of tuberculosis (TB) by 2030, recognize the essential role of tackling latent tuberculosis infection (LTBI) as a strategy for controlling and eliminating TB [1].

The control of LTBI encompasses multiple stages, starting with identifying a population for testing—using the tuberculin skin test (TST) and/or the interferon gamma release assay (IGRA)—and ending with treatment completion [2,3,4]. Several studies have indicated that significant losses occur at all stages of the LTBI cascade, but especially in the early stages and with treatment prescription and adherence to treatment [5].

In the early stages of the TB contact cascade, public health teams need to ensure that all pulmonary TB cases are reported and registered and also that their contacts are identified and registered for screening purposes [2,6]. In addition, protocols need to clearly establish the priority of prescribing treatment to LTBI-positive contacts, while healthcare providers, especially those in primary care, need to be aware of and correctly apply the recommendations of TB elimination guidelines [7,8]. Finally, public health services need to monitor treatment adherence to reduce the risk of new TB cases that could hinder the goal of elimination [9].

Mathematical modelling studies have demonstrated that diagnosing and treating LTBI in people at a high risk of developing active TB, such as contacts, accelerates TB elimination [10,11]. However, the obstacles to the practical implementation of an elimination policy need to be understood. Important information for TB management can be obtained by evaluating the LTBI care cascade and the factors associated with losses at different stages [12].

The aim of the study was to estimate LTBI care cascade losses at three stages, namely, the identification and testing of contacts, treatment prescription, and treatment adherence, in contacts of pulmonary TB cases in Catalonia (Spain).

## 2. Materials and Methods

A prevalence study was conducted on pulmonary TB contacts reported in the 30-month period from 1 January 2019 to 30 June 2021. The inclusion criterion for pulmonary TB cases was having at least one contact who could be identified and screened. The study population consisted of all registered and studied contacts.

Pulmonary TB cases that met the inclusion criteria were epidemiologically surveyed by epidemiology service technicians. All registered contacts were tested (TST and/or IGRA), and also completed a questionnaire on the context and time of exposure to the index case, cohabitation, smoking status, and alcohol-related medical risk (daily intake of >40 g (men) and 24 g (women) or a medical record indicating alcohol abuse).

Contacts who tested positive for IGRA or TST (≥5 mm) were considered to be infected and underwent a posterior–anterior chest X-ray to rule out TB. Sputum samples were obtained from individuals with lesions suggestive of TB to identify acid-fast bacilli and prepare cultures.

We used an LTBI care cascade model, as previously published (12), consisting of the following stages: (1) the identification of close pulmonary TB contacts (reference population); (2) initial screening for LTBI; (3) medical examination including IGRA, TST, and/or X-ray evaluation; (4) recommendation to receive treatment; (5) acceptance and initiation of treatment; and (6) treatment compliance (defined as 80% of the prescribed medication).

Definition of LTBI cascade loss. For the purposes of this study, we considered LTBI cascade losses among the registered participants considered as candidates for initial LTBI screening and also: (1) contacts who failed to show up for testing, test results, or the second TST or IGRA when indicated, or (2) contacts with LTBI who were not prescribed LTBI treatment, or (3) contacts with LTBI who had received a prescription but who failed to treatment compliance.

The LTBI care cascade was studied considering three dependent variables: (1) contacts who were not screened for LTBI, (2) contacts with LTBI who were not prescribed treatment, and (3) contacts with LTBI who were prescribed but failed treatment compliance. Independent variables were age, sex, immigrant status, cohabitation with the index case, the duration of exposure to the index case, tobacco use, alcohol use, exposure to a smear-positive index case, and/or a chest X-ray showing cavernous lesions.

Prevalence rates were calculated as follows: contacts not tested for LTBI; tested contacts who were LTBI-positive; LTBI-positive contacts who received a treatment prescription; and LTBI-positive contacts who adhered to the prescribed treatment. To calculate the cumulative losses at each cascade stage, the proportion remaining in a stage was multiplied by the proportion remaining after the previous stage [13].

Factors associated with treatment prescription and adherence were determined by calculating the odds ratio (OR) and 95% confidence interval (CI). Variables associated with treatment prescription and adherence were calculated as adjusted OR (aOR) and 95% CI values using multivariate logistic regression models developed using the backward stepwise method.

The study was approved by the Ethics Committee of the Arnau Vilanova University Hospital (CEIC-2049) and was conducted according to Declaration of Helsinki principles. All subjects included in the study received detailed information on the study aims before inclusion.

Data collection and cleaning were performed using the Access 12.0 database manager of the MS Office 2013 software package (Microsoft, Redmond, Washington, DC, USA). The analysis was performed using the SPSS v.24 statistical package and the EpiInfo software v7.2.

## 3. Results

Identified from 847 pulmonary TB cases were 7087 contacts, of whom 6537 (92%) could be screened for LTBI. The LTBI prevalence overall was 25.5% (1670/6537), was higher in men than in women (28.5% versus 22.6%; *p* < 0.001), and higher in all age groups compared to children aged <5 years (12.7%; *p* < 0.001).

**Figure 1:**
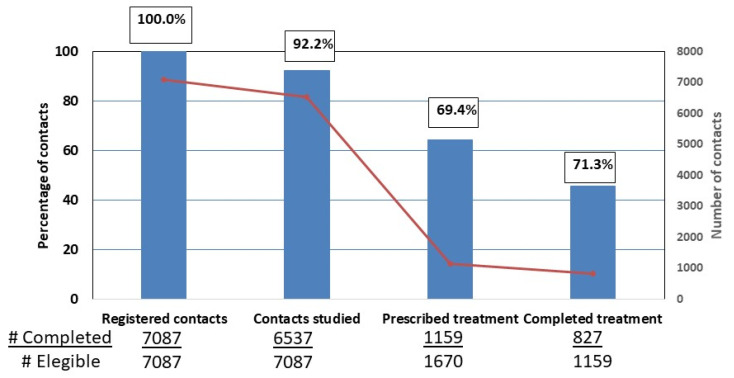
Latent tuberculosis infection care cascade for contacts of pulmonary tuberculosis index cases in a low-incidence region. # number of cases.

Cascade losses were as follows: in the first stage, 7.8% (550/7087), and in the last two stages combined, 50.5% (843/1670), i.e., 30.6% (511/1670) before starting treatment and 19.9% after starting treatment (332/1670). Cumulatively, it was estimated that 45.6% of the contacts would remain in the cascade (Figure 1).

LTBI was studied in lower proportions in persons aged ≥65 years compared to persons aged <18 years (89.2% vs. 93.6%), in men (92.3% vs. 93.8%), and in immigrants (90.9% vs. 93.1%); these differences were statistically significant in the logistic regression model (Table 1).

Treatment was prescribed to 69.4% (1159/1670) of LTBI-positive cases overall; the prescription rate was slightly lower in women (68.6% versus 70.0%) and in the 45–64 (61.8%) and ≥65 (52.3%) age groups. Treatment prescription was higher to people exposed ≥6 h daily (77.7%), people exposed ≥6 h weekly (66.2%), and contacts of positive-smear TB index cases (72.2%). In the multivariate logistic regression model, the variables associated with prescription were the 45–64 (aOR = 0.6; 95%CI: 0.4–0.9) and ≥65 (aOR = 0.3; 95%CI: 0.2–0.6) age groups, ≥6 h daily exposure (aOR = 3.6; 95%CI: 2.6–5.0), ≥6 h weekly exposure (aOR = 2.0; 95%CI: 1.4–2.9), and contacts of positive-smear TB index cases (aOR = 1.3; 95%CI: 1.0–1.7) (Table 2).

Adherence overall was 71.3% (827/1159), and was higher in women (78.0% versus 71.5%) and in the 0–17 (79.1%) and ≥65 (86.7%) age groups. Adherence was lower in immigrants (70.3% versus 79.8%) and in risky alcohol users (72.3% versus 74.4%). In the multivariate logistic regression model, the variables associated with adherence were ages 0–17 (aOR = 1.7; 95%CI: 1.1–2.6) and ≥65 (aOR = 2.5; 95%CI: 1.0–6.3). Immigrant status (aOR = 0.6; 95%CI: 0.4–0.9) and risky alcohol use (aOR = 0.3; 95%CI: 0.2–1.0) were negatively associated with adherence, although the relationship with alcohol was not statistically significant (Table 3).

## 4. Discussion

Our study reveals a substantial loss of patients along the LTBI care cascade. While losses occurred throughout the cascade, the most important losses occurred in the stages before the start of treatment. In the first stage, the 7.8% of lost contacts represent, in absolute terms, a substantial number of people (n = 550). Although many of these contacts may not have been infected, the level of loss suggests the need to strengthen and improve contact studies. In the last two cascade stages, 50.5% of the infected patients were lost (30.6% before and 19.9% after starting treatment). The fact that most losses occurred before starting treatment suggests that the health service prescription of treatment needs to be improved, as concluded in other studies evaluating the LTBI care cascade [5,13].

Persons aged <18 years received the highest proportion of prescriptions, given their higher risk of developing TB once being infected, as evidenced in other studies [14,15]. This age group is therefore a priority in all LTBI control guidelines [4,14,16]. Persons aged >65 years received the lowest proportion of prescription. Some protocols advise against LTBI screening and treatment for this age group, because some infections may not be due to recent exposure and because of possible side effects associated with medication [3,4,13]. Nevertheless, exposure to pulmonary TB cases is clearly high-risk, and consequently, represents an opportunity to prevent possible TB cases and also prevent cascade losses [12,13,17].

Another variable associated with treatment prescription was exposure time to the index case. Since exposure time is associated with the risk of developing LTBI and TB [18], increasing the prescription rate would help to reduce this risk. However, briefer or sporadic but intense exposure (in terms of the volume of air shared with a pulmonary TB case) also entails a high risk [18], so any prescription failures in these cases also represent cascade losses.

Our rate of 71.3% for treatment adherence overall is similar to that reported in other studies [13,19,20,21,22], and was even higher for those aged <18 years, a group at high risk of developing TB [14,15]. The main factors associated with a lower adherence and greater cascade losses were immigrant status and risky alcohol use. Social problems in some groups of immigrants make them more susceptible to treatment non-adherence, as reported by a number of studies [23,24,25,26]. Immigrants thus need to be especially targeted by TB programmes [3,8,27,28], as health provider proactiveness and the direct monitoring of treatment adherence could reduce the corresponding losses in the LTBI care cascade [9]. Although not statistically significant in our logistic regression analysis, risky alcohol use entails an enhanced risk of hepatotoxicity and thus may partially explain treatment non-adherence [2,28,29].

Our results are consistent with the findings of other studies regarding care cascade losses [13,22]. The lower level of prescriptions to older people, associated with insufficient guideline knowledge by healthcare providers, coincides with systematic review observations [13], while other documented reasons for losses include contacts not interested in knowing if they have been infected and contacts perceiving a low risk of infection and disease severity [22].

Public health services need to address the serious health risks associated with TB and the potential sequelae for the respiratory system [23]. Public health teams and community health agents are crucial in order to, first of all, register all cases of pulmonary TB and any contacts that may be candidates for LTBI screening, and then to ensure that LTBI testing is exhaustive [3,8,9]. A key aspect is to identify vulnerable groups at high risk of not contacting the health system. Facilitating access to screening tests, avoiding delays in results, and ensuring social support through community health agents could reduce losses in the early stages of the cascade [30].

Treatment prescription is also crucial to the goal of ultimately eliminating TB, so public health services need to have clear and explicit protocols in place [23,31] and health providers need to scrupulously apply protocol recommendations. Finally, LTBI-positive contacts receiving treatment need to be followed up in TB control programmes to identify and treat possible side effects and ensure adherence.

This study has some limitations. We only included TB cases in which at least one contact was identified. Thus, given that TB cases without contacts could also account for unscreened contacts, losses may be underestimated, although we did not observe this effect. Another possible underestimation arises from the fact that prescription- and treatment-associated factors in the logistic regression models were estimated only for contacts for whom the relevant data were available.

## 5. Conclusions

Our results, pointing to important losses in the LTBI care cascade, suggest a need for patient education, incentives, home visits, and treatment monitoring. The control of LTBI, as key to achieving the goal of ultimately eliminating TB, also requires the implication of the entire health system, but especially of TB clinics [9] and primary care physicians [5]. The inclusion of LTBI data in computerized medical record systems, with primary care protocols and alerts already existing for the control of chronic pathologies, could greatly assist in LTBI monitoring and control.

## Figures and Tables

**Table 1:** Factors associated with latent tuberculosis infection (LTBI) screening of contacts of pulmonary tuberculosis index cases in Catalonia (Spain) (N = 7087).

Variable	LTBI Screening	Univariate Analysis			Adjusted Analysis		
	n/N (%)	OR	95%CI	*p*-Value	aOR	95%CI	*p*-Value
Age, years **							
0–17	1940/2072 (93.6)	1.0	Ref	-	1.0	Ref	-
18–29	1143/1243 (91.9)	0.8	0.6–1.0	0.067	0.9	0.6–1.3	0.629
30–44	1701/1813 (93.8)	1.0	0.8–1.3	0.804	1.0	0.7.–1.4	0.977
45–64	1470/1573 (93.5)	1.0	0.7–1.3	0.829	1.2	0.8–1.6	0.404
≥65	222/249 (89.2)	0.6	0.4–0.9	0.009	0.6	0.4–1.0	0.058
Sex **							
Male	3275/3548 (92.3)	0.8	0.7–0.9	<0.013	0.7	0.6–0.9	0.007
Female	3259/3474 (93.8)	1.0	Ref	-	1.0	Ref	-
Exposure time **							
≥6 h/day	2408/2527 (95,3)	0.8	0.6–1.0	0.066	0.7	0.5–1.1	0.130
<6 h/day but ≥6 h/week	1185/1328 (89.2)	0.3	0.2–0.4	<0.001	0.6	0.4–1.0	0.034
<6 h/week	1952/2024 (96.4)	1.0	Ref	-	1.0	-	-
Sporadic but intense	682/741 (92.0)	0.4	0.3–0.6	<0.001	0.2	−1.9–0.3	<0.001
Immigrant							
Yes	2509/2761 (90.9)	0.7	0.6–0.9	<0.001	1.8	1.4–2.3	<0.001
No	4028/4326 (93.1)	1.0	Ref	-	1.0	Ref	-
Household contact **							
Yes	1957/2058 (95.1)	1.4	1.1–1.8	0.002			
No	4585/4925 (93.1)	1.0	Ref	-			
Smoker							
Yes	845/887 (95.3)	1.8	1.3–2.5	<0.001	1.2	0.8–1.7	0.275
No/unknown	5692/6200 (91.8)	1.0	Ref	-	1.0	Ref	-
Alcohol abuse							
Yes	131/137 (95.6)	1.8	0.8–4.2	0.135	1.2	0.5–2.8	0.697
No/unknown	6406/6950 (92.2)	1.0	Ref	-	1.0	Ref	-
Index case: Sputum smear positive							
Yes	3292/3586 (91.8)	0.9	0.7–1.0	0.163	1.2	1.0–1.6	0.087
No	3245/3501 (92.7)	1.0	Ref	-	1.0	Ref	-
Index case: Rx cavernous lesions							
Yes	2603/2844 (91.5)	0.8	0.7–1.0	0.066	0.7	0.5–0.9	0.004
No	3934/4243 (92.7)	1.0	Ref	-	1.0	Ref	-

Abbreviations: aOR, adjusted odds ratio; CI, confidence interval; OR, odds ratio; *p*-value (chi- square); Ref, reference; Rx, x-ray. ** Missing values (n) variables: Age, years (n = 137); Sex (n = 65); Exposure time (n = 467); and Household contact (n = 104).

**Table 2:** Factors associated with treatment prescription for latent tuberculosis infection (N = 1670).

Variable	Prescription	Univariate Analysis			Adjusted Analysis		
	n/N (%)	OR	95%CI	*p*-Value	aOR	95%CI	*p*-Value
Age, years **							
0–17	259/340 (76.2)	1.0	Ref	-	1.0	Ref	-
18–29	220/280 (78.6)	1.1	0.8–1.7	0.479	1.3	0.9–2.0	0.193
30–44	298/422 (70.6)	0.7	0.5–1.0	0.085	0.8	0.6–1.2	0.265
45–64	328/531 (61.8)	0.5	0.4–0.7	<0.001	0.6	0.4–0.9	0.006
≥65	45/86 (52.3)	0.3	0.2–0.6	<0.001	0.3	0.2–0.6	<0.001
Sex **			0.9–1.3 Ref	0.545			
Male	653/933 (70.0)	1.1			1.1	0.9–1.4	0.367
Female	505/736 (68.6)	1.0			1.0	Ref	-
Exposure time **							
≥6 h/day	700/901 (77.7)	3.3	2.5–4.4	<0.001	3.6	2.6–5.0	<0.001
<6 h/day but ≥6 h/week	194/293 (66.2)	1.8	1.3–2.6	<0.001	2.0	1.4–2.9	<0.001
<6 h/week	139/270 (51.5)	1.0	Ref	-	1.0	Ref	-
Sporadic but intense	92/157 (58.6)	1.3	0.9–2.0	0.155	1.3	0.8–1.9	0.260
Immigrant							
Yes	673/939 (71.7)	1.3	1.0–1.6	0.022	0.6	0.6–1.1	0.122
No	486/731 (66.5)	1.0	Ref	-	1.0	Ref	-
Household contact **				<0.001			
Yes	620/811 (76.5)	2.1	1.1–1.8				
No	462/755 (61.2)	1.0	Ref				
Smoker *				<0.001			
Yes	360/473 (76.1)	1.6	1.2–2.0				
No/unknown	799/1197 (66.7)	1.0	Ref				
Alcohol abuse				0.190			
Yes	47/61 (77.1)	1.5	0.8–2.7		1.1	0.6–2.2	0.663
No/unknown	1112/1609 (69.1)	1.0	Ref		1.0	Ref	-
Index case: Sputum smear positive			1.1–1.7 Ref	0.002			
Yes	749/1038 (72.2)	1.4			1.3	1.0–1.7	0.045
No	410/632 (64.9)	1.0			1.0	Ref	-
Index case: Rx cavernous lesions			1.1–1.6 Ref				
Yes	612/846 (72.3)	1.3		0.066	1.1	0.9–1.5	0.287
No	547/824 (66.4)	1.0		-	1.0	Ref	-

* Abbreviations: aOR, adjusted odds ratio; CI, confidence interval; OR, odds ratio; *p*-value (chi-square); Ref, reference; Rx, x-ray. ** Missing values (n) variables: Age, years (n = 11); Sex (n = 1); Exposure time (n = 49); and Household contact (n = 104).

**Table 3:** Factors associated with treatment adherence for latent tuberculosis infection (N = 1159).

Variable	Adherence	Univariate Analysis			Adjusted Analysis		
	n/N (%)	OR	95%CI	*p*-Value	aOR	95%CI	*p*-Value
Age, years **							
0–17	205/259 (79.1)	1.6	1.1–2.3	0.024	1.7	1.1–2.6	0.038
18–29	157/220 (71.4)	1.0	0.7–1.5	0.889	1.0	0.6–1.4	0.871
30–44	211/298 (70.8)	1.0	Ref	-	1.0	Ref	-
45–64	243/328 (74.1)	1.2	0.8–1.7	0.358	1.1	0.8–1.6	0.616
≥65	39/45 (86.7)	2.7	1.1–6.5	0.031	2.5	1.0–6.3	0.046
Sex **				<0.012			
Male	467/653 (71.5)	0.7	0.5–0.9		0.7	0.5–1.0	0.038
Female	394/505 (78.0)	1.0	Ref		1.00	Ref	-
Exposure time **							
≥6 h/day	498/700 (71.1)	0.8	0.5–1.2	0.379	0.7	0.5–1.2	0.191
<6 h/day but ≥6 h/week	160/194 (82.5)	1.6	0.9–2.7	0.090	1.3	0.7–2.3	0.341
<6 h/week	104/139 (74.8)	1.0	Ref		1.0	Ref	-
Sporadic but intense	72/92 (78.3)	1.2	0.6–2.3	0.548	1.0	0.5–2.0	0.956
Immigrant							
Yes	473/673 (70.3)	0.6	0.4–0.8	<0.001	0.7	0.5–0.9	0.008
No	388/486 (79.8)	1.0	Ref		1.0	Ref	
Household contact **				0.710			
Yes	450/620 (72.6)	0.9	0.7–1.2				
No	340/462 (73.6)	1.0	Ref				
Smoker				0.007			
Yes	286/360 (79.4)	1.5	1.1–2.0				
No/unknown	575/799 (72.0)	1.0	Ref				
Alcohol abuse				0.755			
Yes	34/47 (72.3)	1.1	0.6–2.1		0.3	1.3	0.332
No/unknown	827/1112 (74.4)	1.0	Ref		-	Ref	
Index case: sputum smear positive				0.286			
Yes	564/749 (75.3)	1,2	0.9–1.5		0.9	0.7–1.3	0.651
No	297/410 (72.4)	1.0	Ref		1.0	Ref	
Index case: Rx cavernous lesions				0.013			
Yes	473/612 (77.3)	1.4	1.1–1.8		1.3	1.0–1.3	0.090
No	388/547 (70.9)	1.0	Ref		1.0	Ref	

Abbreviations: aOR, adjusted odds ratio; CI, confidence interval; OR, odds ratio; *p*-value (chi-square); Ref, reference; Rx, x-ray. ** Missing values (n) variables: Age, years (n = 9); Sex (n = 1); Exposure time (n = 34); and Household contact (n = 77).

## Data Availability

The dataset is available from the corresponding author upon reasonable request.
